# Ultra-sonographic and clinical evaluation of ectopic pregnancy among Indian women

**DOI:** 10.6026/9732063002001252

**Published:** 2024-10-31

**Authors:** Afroj A. Khan, Supriya Patil, Rajkumar P. Patange

**Affiliations:** 1 Department of Obstetrics and Gynecology, Krishna Institute of Medical Sciences, Karad, Maharashtra, India - 415110

**Keywords:** Ectopic pregnancy, ultrasonography, post-surgical complications, Clincal aspect, diagnosing

## Abstract

The prevalence of ectopic pregnancy and the risk of mortality from ectopic pregnancy have decreased by 90% in recent years. Therefore, it is of interest to evaluate the use of ultra-sonography and clinical evaluation in diagnosing ectopic pregnancy. Hence, 93 patients were assessed using ultrasonography and clinical aspects. Data shows that majority of patients showed hemo-peritoneum with 73 cases (78.5%) using ultrasonography and abdominal tenderness with 80 cases (86.0%) using clinical evaluation. However, a few patients experienced post-surgical complications like infection.

## Background:

Researchers in their study have been shown that precise diagnosis for pregnant ladies can be done with the help of ultrasonograph [1]. Another study concluded that, the risk of ectopic pregnancy increases in women who have a history of pelvic infections, smoking, impaired fallopian tubes, or who have assisted reproductive procedures. Even in the absence of these risk factors, many women may have an ectopic pregnancy [2]. As a result of the increased availability of transvaginal sonography (TV-S/G) and serum β-HCG testing, ectopic pregnancy was being identified at an earlier and earlier stage [3]. Another study showed that, pelvic ultrasonography have been regarded as the most accurate approach for identify ingectopic pregnancy, since it has changed the diagnostic procedure for ectopic pregnancy and is now considered the most trust-worthy method [4]. In a healthy pregnancy, according to research, there is a link between the levels of β-HCG that are higher than the discriminatory zone, which is the threshold at which an intrauterine gestational sac is anticipated by ultrasonography. The diagnosis of an ectopic pregnancy was made with a hundred percent accuracy when the β-HCG concentration was at least 1,500 IU/l and the uterus was empty during the TV-S/G of the patient [3]. In another study, it is was found that, the early identification of ectopic pregnancy has been significantly assisted by the combination of β-HCG and TV ultrasonography data, which has thus been a significant contribution and it is also possible that the use of color flow Doppler technology(CFDT) will be able to improve diagnostic accuracy even more [5]. A study has shown that, during CFD imaging, ectopic pregnancy often shows a distinct pattern of vessels that are positioned off-center. Other than this, 3D ultrasonography has becoming increasingly popular as a potential supplementary diagnostic technique for ectopic pregnancy. Additionally, they also found that, methotrexate proves to be an effective form of medical treatment in many cases [6]. Studies have also shown that, if a woman is not eligible or has not responded to medicinal therapy with methotrexate then she has a heterotopic pregnancy, or is experiencing hemodynamic instability [3, 4]. According to study, salpingostomy is typically the preferred procedure for women who are still of reproductive age. However, in cases where the fallopian tube is severely damaged, there is a recurring ectopic pregnancy in the same tube, uncontrolled bleeding occurs after salpingostomy, there is a large tubal pregnancy (measuring 5 cm), or the patient has completed their family, salpingectomy may be performed [7]. Therefore, it is of interest to evaluate the role and outcome of ultrasonography and surgical management in diagnosing ectopic pregnancy.

##  Material and Method:

The current prospective interventional study was conducted with 93 patients Department of Obstetrics and Gynecology, KIMS, Karad over a period of 1.5 years starting from June 2022 to Nov 2023 with detailed clinical examination which includes vital signs, abdominal and pelvic examination. Clinical finding includes abdominal pain, vaginal bleeding, amenorrhea, hypotension and tachycardia, adnexal tenderness, cervical motion tenderness, dizziness or syncope and shoulder pain. TV- ultrasonography findings include absence of an intrauterine gestational sac (GS), extra-uterine GS, adnexal mass, tubal ring sign, blob sign, bagel sign and ring of fire sign.

## Inclusion criteria:

>6 weeks of gestation with ectopic pregnancy

## Exclusion criteria:

[1] Intrauterine Gestation

[2] Ectopic pregnancy managed by expectant or medical treatment

## Statistical analysis:

Descriptive statistics was used to summarize the demographic data and clinical characteristics.

## Results:

Table 1 shows that, the majority of cases, 51 (54.8%), fall within the 26-30 years age group. The second largest group is 31-35 years, comprising 18 cases (19.4%). The 21-25 years age group accounts for 16 cases (17.2%). There are fewer cases in the extreme age groups: only 7 cases (7.5%) are over 35 years old and a single case (1.1%) is under 20 years old. This distribution highlights that the majority of the study population is within the 26-30 years age range. Table 2 shows that, among the cases, 37 (39.8%) are primigravida (first pregnancy), while the majority, 56 (60.2%), are multigravida (having had one or more previous pregnancies). Table 3 shows that, the largest group, 44 cases (47.3%), had their last child between 2-5 years ago. This is followed by 27 cases (29.0%) whose last childbirth was 6-10 years ago. There are 13 cases (14.0%) with a last childbirth more than 11 years ago and 9 cases (9.7%) had their last child less than a year ago. Table 4 shows that, the highest proportion of cases, 28 (30.1%), were in the >8 weeks category. This is followed by 22 cases (23.7%) between 7-8 weeks and 43 cases (46.2%) between 6-7 weeks. Table 5 shows that, the most common risk factor identified is tubectomy, present in 20 cases (21.5%). This is followed closely by infertility, with 19 cases (20.4%). Previous LSCS (Lower Segment Cesarean Section) is a factor in 13 cases (14.0%), while a history of abortion is noted in 7 cases (7.5%). Conceiving after ovulation induction and having undergone tuboplasty are each identified in 4 cases (4.3%). Notably, 32 cases (34.4%) have no identifiable risk factor. Table 6 shows that, the most frequently reported symptom is abdominal pain, occurring in 82 cases (88.2%). Amenorrhea is noted in 80 cases (86.0%) and bleeding per vaginam (PV) is seen in 56 cases (60.2%). Vomiting or nausea is present in 20 cases (21.5%), fever in 6 cases (6.5%) and fainting attacks in 2 cases (2.2%). Table 7 shows that, the most common sign is abdominal tenderness, present in 80 cases (86.0%). Cervical motion tenderness is noted in 74 cases (79.6%) and forniceal tenderness is seen in 67 cases (72.0%). Pallor is observed in 45 cases (48.4%), while a mass in the fornix is found in 30 cases (32.3%). Abdominal distension is noted in 22 cases (23.7%) and hypotension and shock, as well as guarding, are present in 13 cases (14.0%) each. Table 8 shows that, the most common finding is a Hemo-peritoneum, observed in 73 cases (78.5%). Adnexa sac without cardiac activity is seen in 68 cases (73.1%). Intrauterine pseudo-gestational sac is noted in 15 cases (16.1%). Lastly, an Adnexal Sac with cardiac activity is identified in 8 cases (8.6%). Table 9 shows that, the majority of cases (91 cases, 97.9%) were tubal pregnancies, with the ampulla being the most common site (73 cases, 78.5%). The isthmus was involved in 11 cases (11.8%), while the interstitial and fimbrial sites each accounted for 4 cases (4.3%). Ovarian ectopic pregnancies were observed in 2 cases (2.2%). Table 10 shows that, out of the total cases, 56 cases (60.2%) hadectopic pregnancy on the right side, while 37 cases (39.8%) had them on the left side.

Table 11 shows that, the modes of termination include tubal rupture in 61 cases (65.6%), tubal abortion in 5 cases (5.4%) and un-ruptured tubal pregnancy in 27 cases (29.0%). Table 12 shows that, in pelvic pathology categories include hydrosalpinx in 11 cases (11.8%), adhesions in 9 cases (9.7%), corpus luteum in 6 cases (6.5%), pelvic haematocele in 4 cases (4.3%) and no pathology identified in 63 cases (67.7%). Table 13 shows that, the majority of cases underwent salpingectomy (removal of the fallopian tube) in 75 instances (80.6%). Less frequently, procedures included salpingo-oophorectomy (removal of the fallopian tube and ovary) in 6 cases (6.5%), milking in 5 (5.4%) and Fimbriectomy in 7 cases (7.5%). Table 14 shows that, 20 cases (21.5%) underwent salpingectomy (removal of the fallopian tube), while salpingo-oophorectomy (removal of the fallopian tube and ovary) was conducted in 4 cases (4.3%). The majority, comprising 69 cases (74.2%), did not require specific treatment for other tube conditions during the study period. Table 15 shows that, out of the total 93 cases analyzed, 35 cases (37.6%) underwent surgery under general anesthesia, while a larger proportion, 58 cases (62.4%), received spinal anesthesia. Table 16 shows that, among the cases reviewed, 4 (4.3%) cases were experienced infections. Interestingly, there were no instances of damage to surrounding organs and anesthesia complications.

## Discussion:

The study was conducted among 93 cases to find out role of ultrasonography in diagnosis of ectopic pregnancy with clinical analysis and management in tertiary care hospital. In present study the majority of cases, 51 (54.8%), fall within the 26-30 years age group. The second largest group is 31-35 years, comprising 18 cases (19.4%). The 21-25 years age group accounts for 16 cases (17.2%). There are fewer cases in the extreme age groups: only 7 cases (7.5%) are over 35 years old and a single case (1.1%) is under 20 years old. This distribution highlights that the majority of the study population is within the 26-30 years age range. In the research conducted by Shetty *et al.* it was shown that the highest number of cases occurred in patients aged 26 to 30 (44%) and 21 to 25 (28%) years. Out of the patients, 16% were over the age of 30, while 12% were below the age of 22 [8]. In present study among the cases, 37 (39.8%) are primigravida (first pregnancy), while the majority, 56 (60.2%), are multigravida (having had one or more previous pregnancies). Study done by Ranji *et al.* found majority of cases (60.5%) were belong to multigravida [9]. The prevalence of ectopic pregnancy was shown to be higher in multiparous women in the many past studies done by Gaddagi *et al.* (62.2%) [10] and Khaleeque *et al.* (61%) [11]. In present study the highest proportion of cases, 28 (30.1%), were in the >8 weeks category. This is followed by 22 cases (23.7%) between 7-8 weeks and 43 cases (46.2%) between 6-7 weeks. Addition to this, most common risk factor identified is tubectomy, present in 20 cases (21.5%). This is followed closely by infertility, with 19 cases (20.4%). Previous LSCS is a factor in 13 cases (14.0%), while a history of abortion is noted in 7 cases (7.5%). Conceiving after ovulation induction and having undergone tuboplasty are each identified in 4 cases (4.3%). Notably, 32 cases (34.4%) have no identifiable risk factors. Our results were similar to Shetty *et al.* results which show that, 2 cases (22%) had a history of tubectomy, 14 cases (14%) had undergone tuboplasty, 6 cases (6%) had a previous ectopic pregnancy and 6 cases (6%) had used an intrauterine contraceptive device. Infertility was noted in 20 cases (20%), a history suggestive of PID in 6 cases (6%) and previous LSCS in 14 cases (14%). A history of abortion was present in 8 cases (8%), while 4 cases (4%) conceived after ovulation induction. Notably, 34 cases (34%) had no identifiable risk factors. Kostrzewa *et al.* found that the recurrent risk of ectopic pregnancy was 19.4% after salpingectomy and 13.6% with salpingotomy, based on a 24-month follow-up of women's fertility after surgical treatment of tubal ectopic pregnancy [12]. The Refaat *et al.* review found when a woman with an in situ IUCD misses her period, it is important to closely monitor her for an ectopic pregnancy [13]. A multicenter, case-control research carried out in China came to the conclusion that IVF-ET and contemporary IUCD usage, in addition to the usual risk factors, are major contributors to the incidence of ectopic pregnancy [14].

The most frequently reported symptom is abdominal pain occurring in 82 cases (88.2%). Amenorrhea is noted in 80 cases (86.0%) and bleeding per vaginam (PV) is seen in 56 cases (60.2%). Vomiting or nausea is present in 20 cases (21.5%), fever in 6 cases (6.5%) and fainting attacks in 2 cases (2.2%). Furthermore, most common sign is abdominal tenderness, present in 80 cases (86.0%). Cervical motion tenderness is noted in 74 cases (79.6%) and forniceal tenderness is seen in 67 cases (72.0%). Pallor is observed in 45 cases (48.4%), while a mass in the fornix is found in 30 cases (32.3%). Abdominal distension is noted in 22 cases (23.7%) and hypotension and shock, as well as guarding, are present in 13 cases (14.0%) each. Additionally, most common finding is a Hemo-peritoneum, observed in 73 cases (78.5%). Adnexa sac without cardiac activity is seen in 68 cases (73.1%). Intrauterine pseudo-gestational sac is noted in 15 cases (16.1%) Lastly, an Adnexal Sac with cardiac activity is identified in 8 cases (8.6%). In our study, we also found that, the majority of cases (91 cases, 97.9%) were tubal pregnancies, with the ampulla being the most common site (73 cases, 78.5%). The isthmus was involved in 11 cases (11.8%), while the interstitial and fimbrial sites each accounted for 4 cases (4.3%). Ovarian ectopic pregnancies were observed in 2 cases (2.2%). According to Shetty, the ampulla of the fallopian tube was the most often found location of the ectopic pregnancy, accounting for 45.2% of cases [15]. Gaddagi *et al.* reported similar results, *i.e.*, ampulla pregnancies accounted for the majority of instances (69.7%) [10].

In present study out of the total cases, 56 cases (60.2%) had ectopic pregnancies on the right side, while 37 cases (39.8%) had them on the left side. While the modes of termination include tubal rupture in 61 cases (65.6%), tubal abortion in 5 cases (5.4%) and unruptured tubal pregnancy in 27 cases (29.0%). In the Chate *et al.* study, the incidence of rupture was 76.35%. Tubal abortion was seen in 16.12%, followed by unruptured ectopic pregnancies at 7.53% [16]. Gaddadi *et al.* reported similar findings, with 78.3% of patients having a ruptured ectopic pregnancy after laparotomy [10]. Tubal abortion occurred in four cases, whereas three cases involved an unruptured ectopic pregnancy. Shetty *et al.* reported unruptured ectopic and tubal abortions in 12.9% of patients [15].

In present study pelvic pathology categories include hydrosalpinx in 11 cases (11.8%), adhesions in 9 cases (9.7%), corpus luteum in 6 cases (6.5%), pelvic haematocele in 4 cases (4.3%) and no pathology identified in 63 cases (67.7%). On the other hand, majority of cases underwent salpingectomy (removal of the fallopian tube) in 75 instances (80.6%). Less frequently, procedures included salpingo- oophorectomy (removal of the fallopian tube and ovary) in 6 cases (6.5%), milking in 5 (5.4%) and Fimbriectomy in 7 cases (7.5%). Megier *et al.* studied 100 colour and pulsed Doppler examinations of tubal ectopic pregnancies and discovered that colour Doppler can help diagnose tiny ectopic pregnancies (gestational sacs < 1 cm and echogenic adnexal masses < 2 cm) with high impedence flow (diastolic index < 0.35) [17]. Another study disclosed a novel Doppler ultrasonography sign known as the "leash sign" with 100% sensitivity, 99% specificity and 95% PPV and 100% NPV [7].

## Conclusion:

There was a high incidence of tubal rupture and tubal abortion, while most of the cases were managed through salpingectomy. Trans-vaginal/ultra-sonography is a highly reliable method for diagnosing ectopic pregnancy. The findings highlight the importance of early detection and comprehensive management to mitigate the adverse outcomes associated with ectopic pregnancies.

## Figures and Tables

**Table 1:** Age distribution

**Age group**	**Cases**	**Percent**
< 20 years	1	1.10%
21-25 years	16	17.20%
26-30 years	51	54.80%
31-35 years	18	19.40%
> 35 years	7	7.50%
Total	93	100%

**Table 2:** Gravida distribution

**Gravida**	**Cases**	**Percent**
Primi	37	39.80%
Multi	56	60.20%
Total	93	100%

**Table 3:** Last child birth

**Last child birth**	**Cases**	**Percent**
<1year	9	9.70%
2-5 years	44	47.30%
6-10 years	27	29.00%
>11 years	13	14.00%
Total	93	100%

**Table 4:** GA distribution

**Gestation age (GA)**	**Cases**	**Percent**
6-7weeks	43	46.20%
7-8weeks	22	23.70%
> 8weeks	28	30.10%
Total	93	100%

**Table 5:** R/F

**Risk Factors (R/F)**	**Cases**	**Percent**
Tubectomy	20	21.50%
Infertility	19	20.40%
Previous LSCS	13	14.00%
H/O abortion	7	7.50%
H/O previous ectopic pregnancy	6	6.50%
Intrauterine contraceptive device (IUCD)	6	6.50%
History suggestive of PID	6	6.50%
Conceived after ovulation induction	4	4.30%
Tuboplasty	4	4.30%
No identifiable risk factors	32	34.40%

**Table 6:** Symptom distribution

**Symptoms**	**Cases**	**%**
Pain abdomen	82	88.20%
Amenorrhea	80	86.00%
Bleeding PV	56	60.20%
Vomiting/Nausea	20	21.50%
Fever	6	6.50%
Fainting attacks	2	2.20%

**Table 7:** Sign distribution

**Signs**	**Cases**	**%**
Abdominal tenderness	80	86.00%
Cervical motion tenderness	74	79.60%
Fornicial tenderness	67	72.00%
Pallor	45	48.40%
Mass in the fornix	30	32.30%
Distension	22	23.70%
Hypotension and shock	13	14.00%
Guarding	13	14.00%

**Table 8:** U/S finding

**Ultra-sonographic Findings**	**Cases**	**%**
Hemoperitoneum	73	78.50%
Adnexal sac with cardiac activity	8	8.60%
Adnexal sac without cardiac activity	68	73.10%
Intrauterine pseudo-gestational sac	15	16.10%

**Table 9:** Laparoscopic distribution

**Laparoscopic / Laparotomy findings: Site**	**Cases**	**%**
Tubal (A)	91	97.90%
Ampulla	73	78.50%
Isthmus	11	11.80%
Interstitial	4	4.30%
Fimbrial	4	4.30%
Ovary (B)	2	2.20%
Total (A+B+C)	93	100%

**Table 10:** Side

**Laparoscopic / Laparotomy findings: Side**	**Cases**	**%**
Right	56	60.20%
Left	37	39.80%
Total	93	100%

**Table 11:** P Distribution

**Mode of termination tubal pregnancy (TP)**	**Cases**	**%**
Tubal rupture	61	65.60%
Tubal abortion	5	5.40%
Unruptured	27	29.00%
Total	93	100%

**Table 12:** Pelvic pathology

**Pelvic pathology**	**Cases**	**%**
Hydrosalpinx	11	11.80%
Adhesions	9	9.70%
Corpus luteum	6	6.50%
Pelvic haematocele	4	4.30%
No pathology	63	67.70%
Total	30	100%

**Table 13:** Ectopic distribution

**Treatment for Ectopic**	**Cases**	**%**
Salpingectomy	75	80.60%
Salpingo-oophorectomy	6	6.50%
Milking	5	5.40%
Fimbriectomy	7	7.50%
Total	93	100%

**Table 14:** Tube treatment

**Treatment for Other Tube**	**Cases**	**%**
Salpingectomy	20	21.50%
Salpingo-oophorectomy	4	4.30%
No treatment for other tube	69	74.20%
Total	93	100%

**Table 15:** Anaesthesia used

**Type of Anaesthesia used**	**Cases**	**%**
General	35	37.60%
Spinal	58	62.40%
Total	93	100%

**Table 16:** Complication

**Immediate & short-term Complications**	**Cases**	**Percentage**
Infection	4	4.30%
Damage to surrounding Organ	0	0.00%
Anaesthesia Complications	0	0.00%
